# Supplementary material for: Improvement of Peptidyl Copper Complexes Mimicking Catalase: A Subtle Balance between Thermodynamic Stability and Resistance towards H2O2 Degradation
Source: Molecules. 2022 Aug 26;27(17):5476. doi: 10.3390/molecules27175476 (PMC9457919; doi:10.3390/molecules27175476)
Supplement: Supplementary file 1 [file molecules-27-05476-s001.zip › molecules-1847795-supplementary.pdf]

Improvement of peptidyl copper complexes mimicking Catalase: a subtle balance between thermodynamic stability and resistance towards H_2O_2 degradation

Yaqine Ben Hadj Hammouda, Koudedja Coulibaly, Alimatou Bathily, Magdalene Teoh Sook Han, Clotilde Policar and Nicolas Delsuc*

Laboratoire des biomolécules, LBM, Département de chimie, Ecole normale supérieure, PSL University, Sorbonne Université, CNRS, 75005 Paris, France

Outline

1. Supplementary figures and tables 2
2. Chemicals and instruments 5
3. Procedures 6
4. References 7

1. Supplementary figures and tables

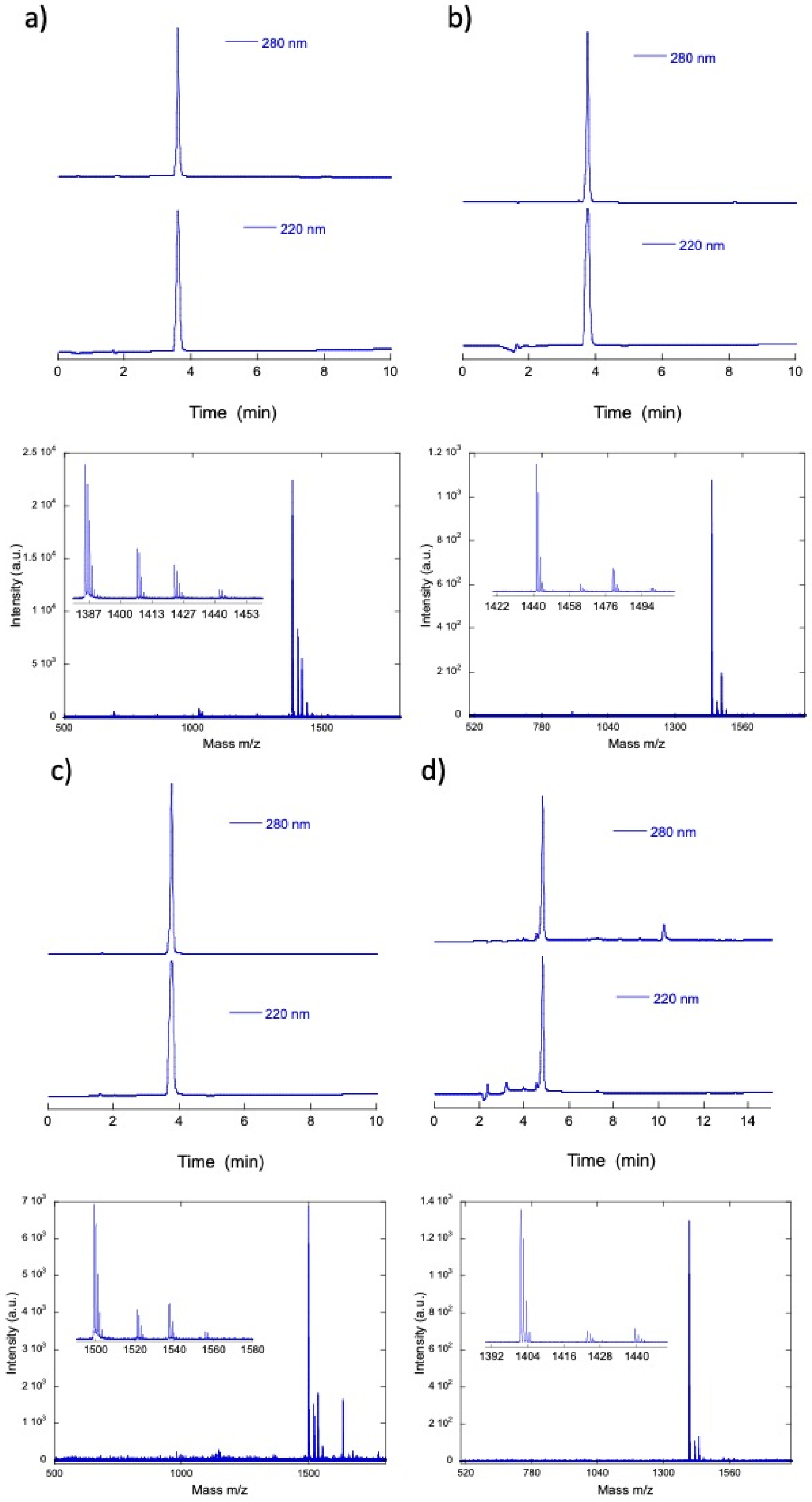

Figure S1. Characterization of the synthesized peptides a) CATm2, b) CATm3, c) CATm4 and d) CATm5. The top figures are the HPLC traces recorded at 280 and 220 nm and bottom figures are the MALDI-TOF mass spectra of the peptides. The insets are zooms of the region of interest.

Table S1. Characterization of the newly synthesized peptide.

Name	Sequence	Expected Mass $[M+H]^+$	Found Mass $[M+H]^+$	Retention time (min)
CATm2	Ac-PHYKHPHYKH-NH_2	1384.68	1384.9	3.61[a]
CATm3	Ac-PHYKHGPHYKH-NH_2	1441.71	1441.6	3.76[a]
CATm4	Ac-PHYKHGGPHYKH-NH_2	1498.73	1499.0	3.77[a]
CATm5	Ac-PHYKHGGHYKH-NH_2	1401.67	1401.6	4.84[b]

[a] 5 to 95% CH_3CN in H_2O both containing 0.1% TFA using a Proto 200 C18 3 µm Higgins Analytical Inc. 100 x 4.6 mm column
[b] 5 to 100% CH_3CN in H_2O both containing 0.1% TFA using or a Nucleodur C18 100-3 ec Macherey-Nagel 150 x 4.6 mm column.

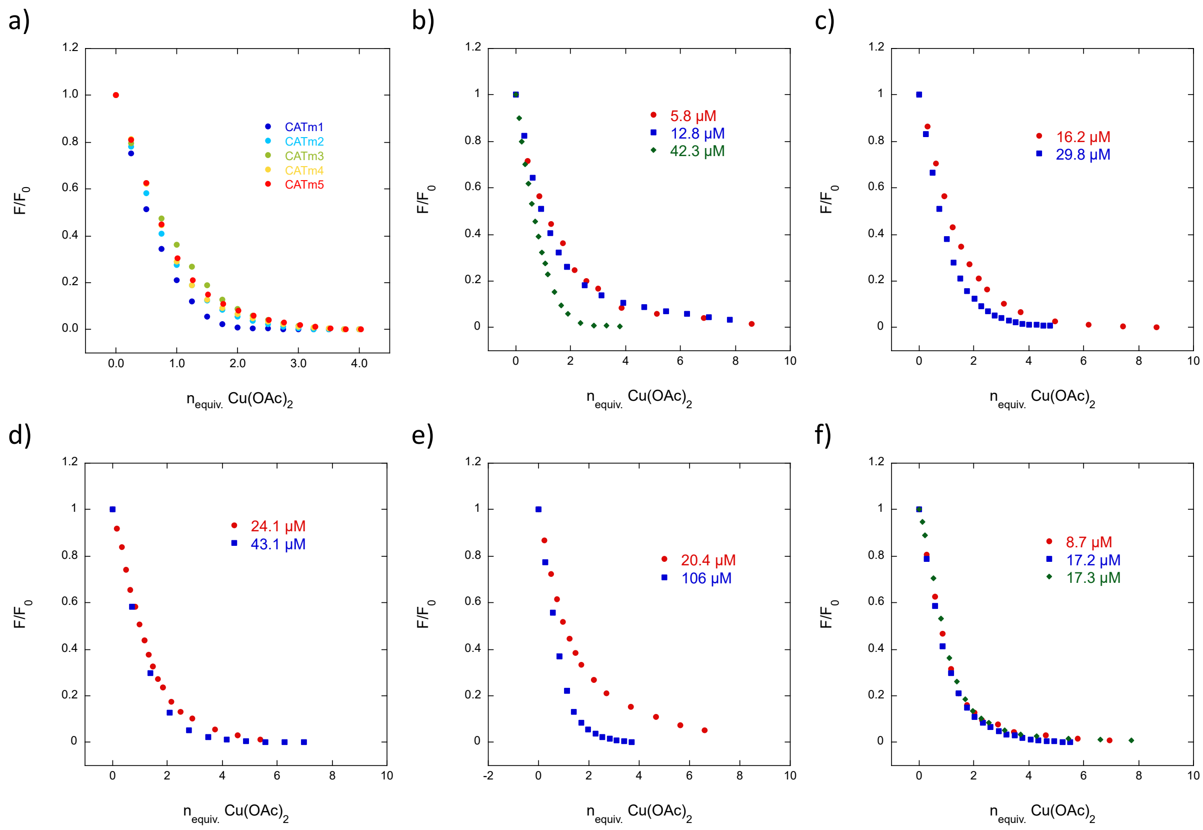

Figure S2. $Cu(OAc)_2 \bullet H_2O$ titration into CATmx peptide solution in MOPS buffer (50 mM, pH 7.5) at 25 °C. Spectra were recorded upon irradiation of tyrosine at 275 nm and the emission at 303 nm was measured after each addition. a) The concentration of the peptides was set between 72 to 75 µM. b) Titrations involving CATm1. c) Titrations involving CATm2. d) Titrations involving CATm3. e) Titrations involving CATm4. f) Titration involving CATm5.

Table S2. Apparent association constants of the two binding sites for each complex measured using fluorescence spectroscopy at 25°C in MOPS buffer (50 mM, pH 7.5). Excitation was set at 275 nm and spectra were recorded from 280 to 400 nm.

	${}^{app}K_{a1}$ (10^6)	${}^{app}K_{a2}$ (10^6)
CATm1:Cu 1:2	0.361 ± 0.005	0.118 ± 0.005
CATm2:Cu 1:2	29.0 ± 0.8	0.166 ± 0.002
CATm3:Cu 1:2	0.154 ± 0.005	0.091 ± 0.002
CATm4:Cu 1:2	3.26 ± 0.09	0.076 ± 0.002
CATm5:Cu 1:2	48.7 ± 3.5	0.3351 ± 0.0006

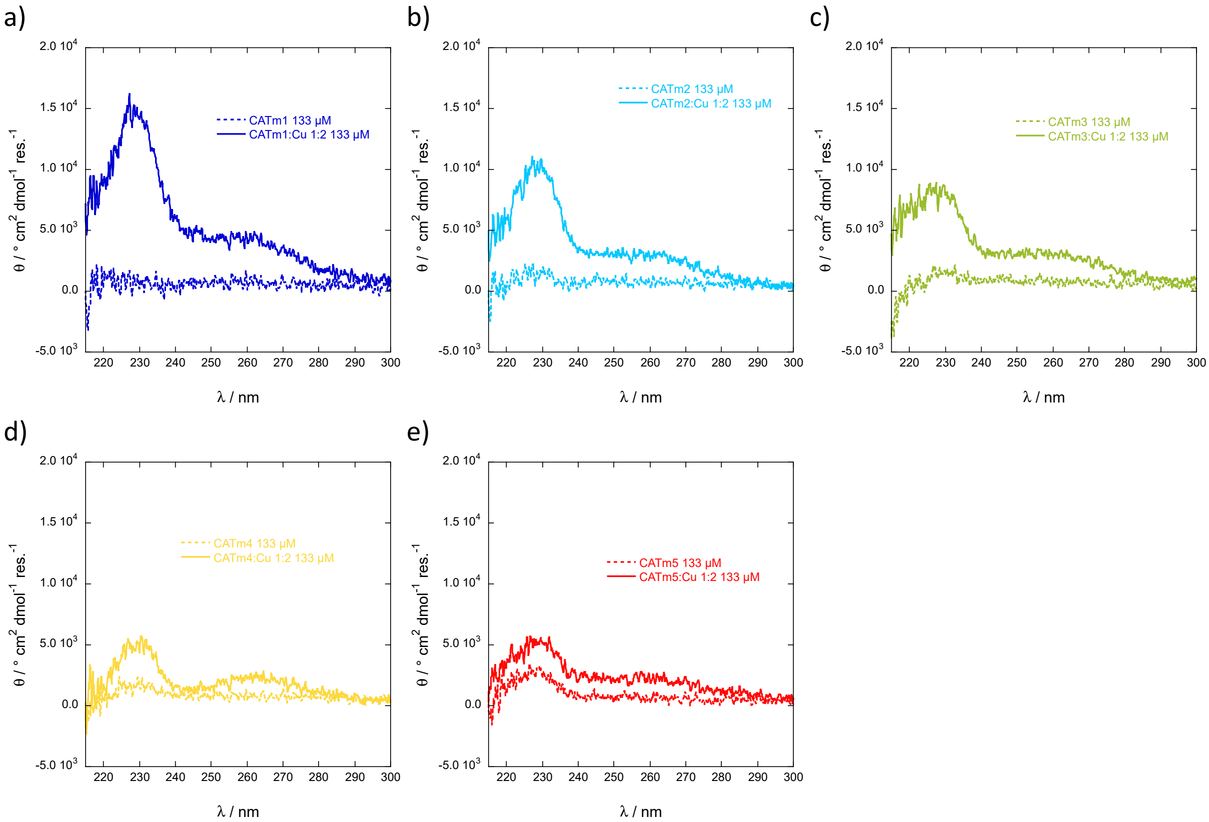

Figure S3. Circular dichroism spectra of CATmx (dashed lines) and CATmx:Cu 1:2 mixtures (solid lines) at 133 µM (x = 1-5). Spectra were recorded at 20°C in MOPS buffer (50 mM, pH 7.5). a) CATm1, b) CATm2, c) CATm3, d) CATm4 and e) CATm5.

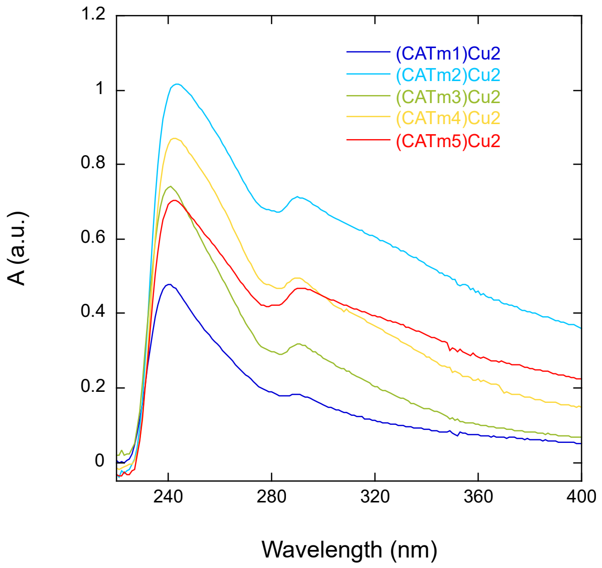

Figure S4. Subtracted UV-vis spectra (after - before addition of a large excess of H_2O_2) of CATmx:Cu 1:2 mixtures (x = 1-5). All the products resulting from degradation exhibit two similar main absorbance bands at 240 and 289 nm.

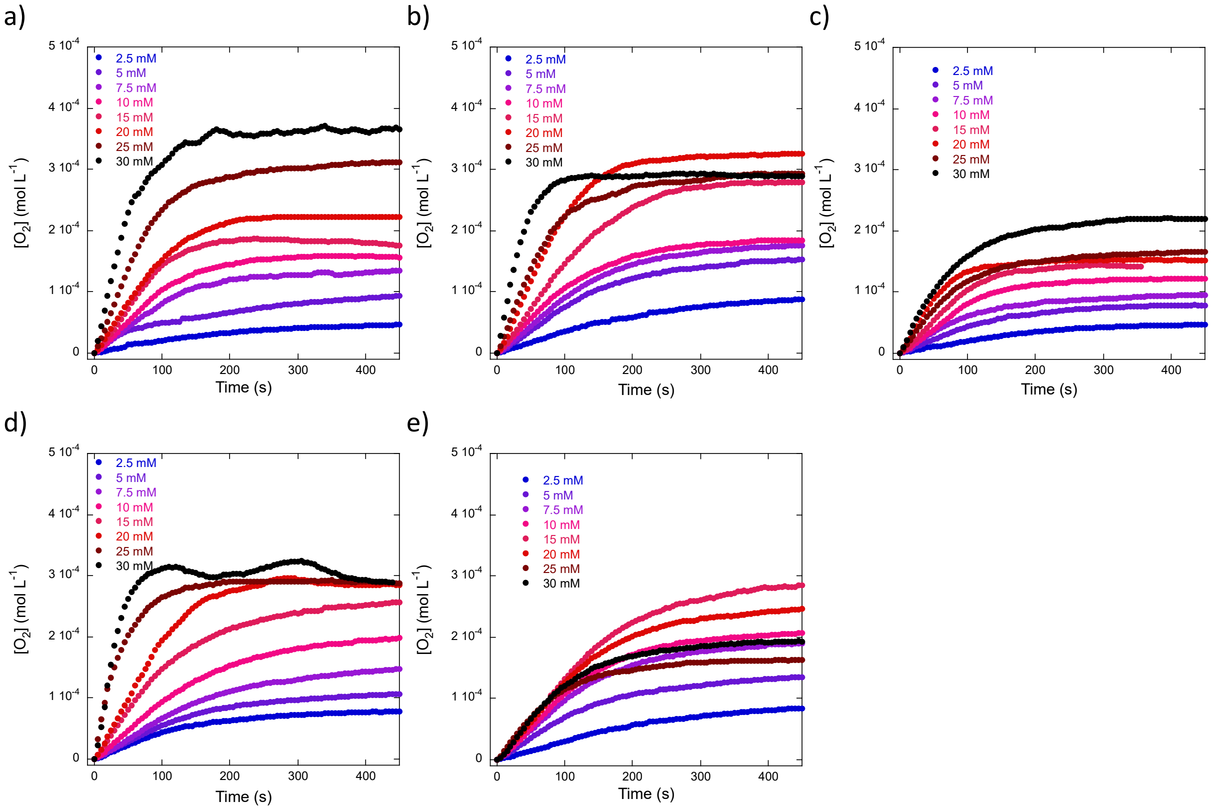

Figure S5. Dioxygen evolution monitored using a Clark-type electrode of CATmx:Cu 1:2 mixtures (x = 1-5) at 100 µM in MOPS buffer (50 mM, pH 7.5) at 25°C in presence of increasing concentrations of [H_2O_2] (from 2.5 mM to 30 mM). a) (CATm1)Cu_2, b) (CATm2)Cu_2, c) (CATm3)Cu_2, d) (CATm4)Cu_2, e) (CATm5)Cu_2.

2. Chemicals and instruments

All chemicals and solvents were of synthesis grade and were used as received without further purification. Common solvents for solid support synthesis and L-amino acids were obtained from either Sigma Aldrich, Novabiochem or Iris biotech GMBH. MOPS buffer was prepared using the MOPS salt from Sigma Aldrich and the pH was adjusted at 7.5.

Instruments

- MALDI-TOF mass spectra were recorded on a Voyager DE-Pro MALDI-TOF mass spectrometer (Applied Biosystems) in positive mode using as matrix a solution of α- Cyano-4-hydroxycinnamic acid (CHCA) at 10 mg/mL in CH_3CN:H_2O (50:50) containing 0.1% TFA. Calibration was performed using external standards (Proteomix 4, LaserBio Labs Sofia-Antipolis, France).

- Analytical HPLC was performed on an Agilent Technologies 1200 Series equipped with a multiple wavelength absorbance detector, using a Proto 200 C18 3 µm Higgins Analytical Inc. 100 x 4.6 mm column or a Nucleodur C18 100-3 ec Macherey-Nagel 150 x 4.6 mm column.

- Preparative HPLC was performed on a Waters 600 HPLC Pump equipped with a Waters 2487 dual wavelength absorbance detector, using a Column VP 260/16 C18 Hter 5 µm

- UV-vis spectrometry was performed on a Cary 300 bio in a double beam mode with the buffer in the reference cell at 25°C.

- Circular dichroism (CD) spectra (215-300 nm) were collected on a J-810 spectropolarimeter (Jasco, Tokyo, Japan) at 20°C. The scan rate, the sensitivity, and bandwidth

were 200 nm.min^{-1}, 0.125 s, and 1 nm respectively. Each spectrum was an average of five scans.

- Kinetics analysis using Clark-type electrode: the ability of the complex to catalyze H_2O_2 was measured by polarimetry. An O_2-sensitive Clark-type electrode OD InLab 605-ISM purchased from Mettler Toledo was used. Prior to measurements, the electrode was calibrated in ambient air then in a provided 0% dissolved oxygen solution.

3. Procedures

Kinetic study of complex degradation by UV-visible spectroscopy

Molar extinction coefficient of degradation product determination. For (CATm1)Cu_2, the previously reported value rate for degradation (2.7 10^{-3} s^{-1}) [1] was obtained by determining ε from the UV spectra obtained at the end of the experiment and considering that no more intact catalyst remained. In order to insure a better accuracy of the results, we have calculate the rates by measuring ε as follows:

H_2O_2 in excess was added to a solution CATmx:Cu 1:2 (x = 1-5) of complex (500 µM:1 mM, 1 mL) (50 mM). This solution was kept for 24h to ensure the total formation of the degradation product. Then, the solution was diluted with MOPS buffer (50 mM, pH 7.5) to obtain 4 solutions of complex concentration ranging from 25 to 125 µM. The absorbance was measured from 600 nm to 200 nm and the values at 289 nm were extracted and plotted as a function of complex concentration, to provide the molar extinction coefficient.

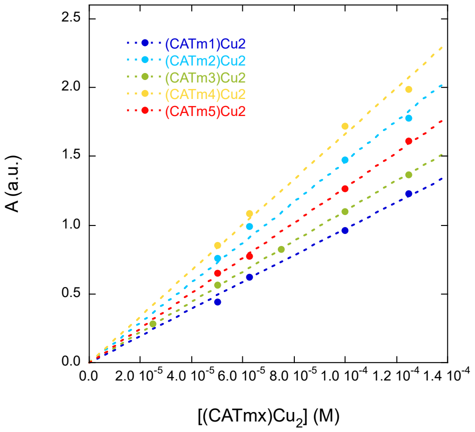

Figure S6. Molar extinction coefficient (ε) determination of the degradation product of CATmx:Cu(OAc)$_2$ 1:2 (x =1 to 5). Excess H_2O_2 (50 mM) was added to initial solutions of complex at different concentrations (25, 50, 62.5, 100 and 125 µM) in MOPS (50 mM, pH 7.5). The absorbance at 289 nm of this solution was measured for the different concentrations and enabled the determination of ε.

Kinetics experiments. Complex (CATmx:Cu 1:2) degradation kinetics was monitored for each complex (x = 1-5) at 4 different concentrations (20, 50, 100 and 200 µM) in MOPS buffer (50 mM, pH 7.5). The absorbance was recorded at 289 nm over time, until a plateau was reached. The addition of H_2O_2 in excess (5 mM) into the cuvette corresponds to the beginning of the experiment (t0). The initial rates of degradation product formation v_0 were measured from the slope of linear fits (from 0 to 30 s) for the different complex concentrations. For each complex concentration, the measurements were performed twice and the values used to plot the figure 3 were average ± standard error of the mean (SEM). Then, k_{obs} corresponds to the slope of the linear fit of v_0 = f([complex]).

4. References

1. Coulibaly, K.; Thauvin, M.; Melenbacher, A.; Testard, C.; Trigoni, E.; Vincent, A.; Stillman, M.J.; Vriz, S.; Policar, C.; Delsuc, N. A Di-Copper Peptidyl Complex Mimics the Activity of Catalase, a Key Antioxidant Metalloenzyme. *Inorg. Chem.* **2021**, *60*, 9309–9319, doi:10.1021/acs.inorgchem.0c03718.
